# Oxidative Stress as a Central Mechanistic Bridge Between Alzheimer’s and Vascular Pathologies in Mixed Dementia: Emerging Evidence and Therapeutic Perspectives

**DOI:** 10.3390/biomedicines14010059

**Published:** 2025-12-26

**Authors:** Francesca Beretti, Marta Malenchini, Martina Gatti, Tullia Maraldi

**Affiliations:** 1 Department of Biomedical, Metabolic and Neural Sciences, University of Modena and Reggio Emilia, 41125 Modena, Italy; 2 Cellular Signalling Laboratory, Department of Biomedical and Neuromotor Science, University of Bologna, 401126 Bologna, Italy

**Keywords:** oxidative stress, mixed dementia, extracellular vesicles

## Abstract

Mixed dementia (MD), characterized by overlapping features of Alzheimer’s disease (AD) and vascular dementia (VaD), represents the most prevalent form of late-life cognitive decline. Increasing evidence identifies oxidative stress as a unifying molecular mechanism driving both neurodegenerative and vascular pathologies in MD. Reactive oxygen species (ROS) contribute to amyloid-β aggregation, tau hyperphosphorylation, endothelial dysfunction, and blood–brain barrier disruption, creating a self-perpetuating cycle of neuronal and vascular injury. Mechanistic models demonstrate how chronic hypoperfusion and mitochondrial dysfunction exacerbate ROS generation and neuroinflammation, while impaired Nrf2-mediated antioxidant defense further amplifies damage. Therapeutically, classical antioxidants show inconsistent efficacy, shifting focus toward mitochondrial protection, Nrf2 activation, and lifestyle-based oxidative load reduction. Therefore, we sought to outline therapeutic approaches capable of broadly targeting these mechanisms, through focused narrative analysis of recent studies employing delivery systems for antioxidant proteins and/or redox-regulating miRNAs. In particular, experimental interventions using mesenchymal stem cell-derived extracellular vesicles (MSC-EVs) demonstrate neuroprotective and anti-inflammatory effects via the Nrf2 pathway, suggesting promising avenues for multimodal treatment. Integrating oxidative, vascular, and neurodegenerative paradigms is essential for advancing diagnostic precision and developing targeted interventions capable of addressing the complex pathophysiology of mixed dementia.

## 1. Introduction

### 1.1. Alzheimer and Vascular Dementia Combine in Mixed Dementia

Dementia is a leading cause of disability and dependency among older adults worldwide, with Alzheimer’s disease (AD) and vascular dementia (VaD) representing the two most prevalent forms. Increasing evidence indicates that these conditions often coexist as mixed dementia (MD), particularly in the elderly population, where overlapping neurodegenerative and cerebrovascular pathologies contribute synergistically to cognitive decline [1,2]. This coexistence complicates diagnosis and treatment, as the clinical manifestations reflect both amyloid-driven neurodegeneration and ischemic white matter injury.

The risk factors for cerebrovascular diseases and AD are diverse, often co-occur, and raise the question of whether dementias involving vascular processes are profoundly distinct from those caused by the accumulation of amyloid-β 42 and tau proteins or whether both pathological mechanisms have an additive impact on cognitive activity [3]. Individuals with AD dementia often exhibit autopsy evidence of mixed brain pathologies, including a myriad of vascular changes, cerebrovascular injuries, complex brain inflammation, and mixed protein inclusions in addition to hallmark neuropathologic lesions of AD, namely insoluble amyloid-β (Aβ) plaques and neurofibrillary tangles (NFTs). Epidemiological data demonstrate that the coexistence of these lesions lowers the Aβ plaque and NFT threshold necessary to precipitate clinical dementia [4].

A major pathological contributor to vascular cognitive impairment (VCI) and MD is chronic cerebral hypoperfusion, which leads to white matter lesions, neuronal loss, and microvascular dysfunction. Vascular risk factors cause blood–brain barrier (BBB) impairment and a decreased cerebral blood flow, leading to a reduced brain tissue perfusion. Disruption of the BBB—as evidenced by increased permeability measured via dynamic contrast-enhanced MRI—has been directly associated with white matter injury and cognitive impairment in both aging and neurodegenerative diseases [5]. Primarily, BBB dysfunction induces the accumulation of neurotoxic compounds, which are linked to the production of numerous focal ischemic infarcts and microinjuries caused by hypoxia, resulting in neuronal damage. Vascular injury increases amyloid precursor protein (APP) expression and its processing along the amyloidogenic pathway, raising the Aβ-peptide level. Additionally, BBB injury impairs Aβ peptide clearance, further contributing to amyloid accumulation. Such deposition in the brain exacerbates neuronal malfunctioning, hastening the progression of neurodegeneration. Moreover, the Tau protein is hyperphosphorylated due to both ATP imbalance and hypoperfusion, which helps generate NFT. Supporting this, elevated serum zonulin, a regulator of tight junction integrity, has been observed in patients with AD and MD but not in VaD, indicating early barrier dysfunction specific to amyloid-related pathology [6].

Despite different points of view, through both amyloidogenic and non-amyloidogenic routes, events of BBB dysfunction cause neural destruction.

Beyond vascular factors, oxidative stress and neuroinflammation are emerging as central pathogenic mechanisms bridging AD, VaD, and MD.

### 1.2. Oxidative and Inflammatory Dysregulation in Dementia

Clinical analyses have demonstrated elevated levels of reactive oxygen metabolites and reduced antioxidant capacity in patients with AD and MD compared to non-demented controls, suggesting an imbalance between pro-oxidant and antioxidant systems [7].

**Figure 1:**
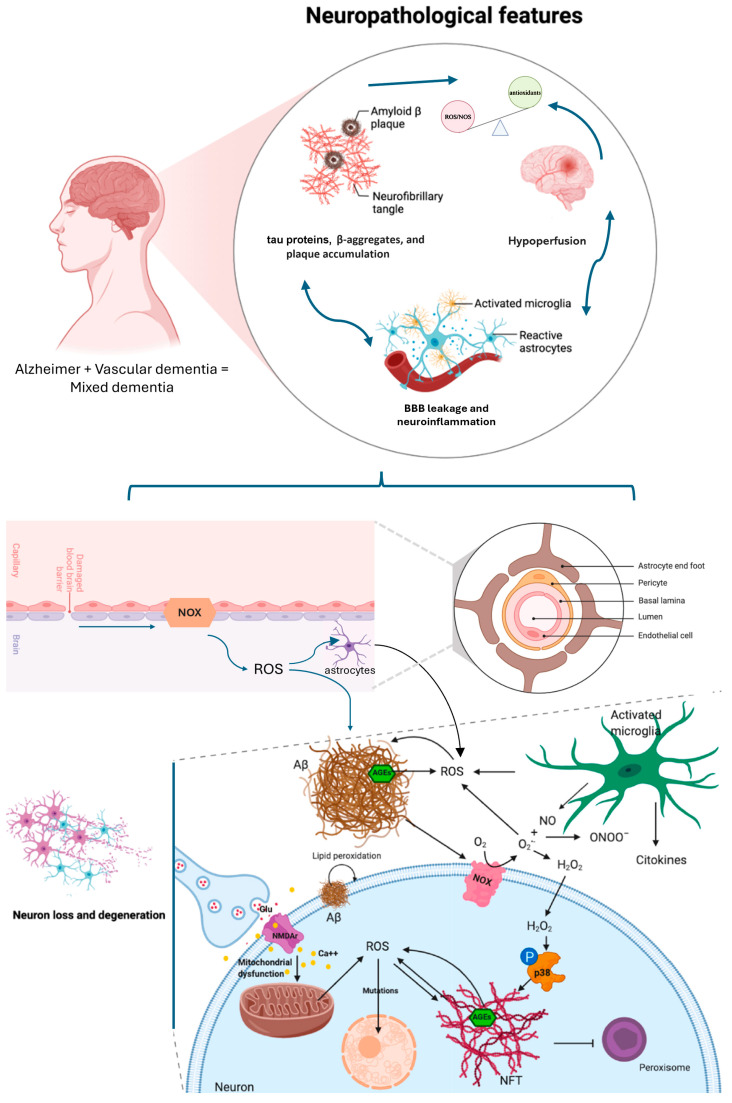
Scheme of neuropathological features occurring in Alzheimer Disease and in Vascular Dementia, driving mixed dementia. The focus is on the redox imbalance shared by these diseases and that potentiates the pathogenetic pathway affecting neuron viability. The blue color was used to better differentiate the context of brain barrier and the interstitial space between neurons and glial cells (black color). Modified from Angeloni et al., 2020 [8].

A schematic representation of causes and consequences of oxidative stress in the brain is shown in Figure 1. ROS can be produced by mitochondria, or by the reactions catalyzed by NADPH oxidases or nitric oxide synthase (NOS). Oxidative stress can also be induced by reactive nitrogen species (RNS), by the fast reaction of O2•− with nitric oxide (NO), resulting in the generation of peroxynitrite (ONOO−) that can trigger DNA fragmentation and lipid peroxidation. Cells respond to oxidative stress by inducing antioxidant enzymes that can neutralize ROS counteracting cell damage. Nuclear factor erythroid 2-related factor 2 (Nrf2) enhances the expression of these antioxidant enzymes thanks to its binding to a specific sequence in the promoter region of these genes called antioxidant response element (ARE). In normal physiological condition, Nrf2 is sequestered in the cytoplasm by Kelch-Like ECH-Associated Protein 1 (Keap1) that promotes ubiquitination and degradation of Nrf2 in the proteasome. Nevertheless, when ROS or electrophiles modify the nucleophilic cysteine sulfhydryl groups on Keap1, Nrf2 is released and it moves to the nucleus. Furthermore, Nrf2 also exerts anti-inflammatory activity and modulates both biogenesis and mitochondrial function. On these bases, Nrf2 is considered an interesting therapeutic target in counteracting neurodegeneration [8].

The newest evidence continues to support oxidative stress as a shared, central mechanism connecting the vascular and neurodegenerative components of mixed dementia—with one human study explicitly flagging a higher mixed dementia risk tied to a genotype that impairs resilience to oxidative stress [9]. Lyu in 2024 [10] highlighted mitochondrial dysfunction and impaired mitophagy as upstream sources of ROS implicated across neurodegenerative and vascular cognitive disorders—again fitting mixed dementia’s dual pathology. Another work of 2024 [11] synthesizes how chronic hypoperfusion/hypoxia triggers a vicious cycle of oxidative stress and neuroinflammation that propagates vascular injury and neurodegeneration—exactly the bidirectional feed-forward loop expected in mixed dementia. The extent of oxidative stress-related damage may differ across the subtypes of dementia by being greater in the MD group than in other types of dementia. Synergic effects of the AD degenerative pathology and white matter lesions might be associated with oxidative stress damage in the MD group [7]. Both amyloid/tau pathology (AD) and chronic cerebral hypoperfusion/small-vessel disease (vascular component) increase ROS, neuroinflammation and proteotoxic stress—all canonical activators/clients of the Nrf2 system. However, aging and disease processes often produce Nrf2 dysfunction, impairing the endogenous antioxidant response and permitting accumulation of oxidative damage [12,13]. This is a core reason Nrf2 is centrally relevant in mixed dementia. Here we summarize oxidative stress markers that are elevated in these pathologies (Table 1).

Indeed, parallel to oxidative mechanisms, chronic low-grade inflammation (“inflammaging”) has been implicated in cognitive decline. In older adults, inflammatory markers such as interleukin-1β, interleukin-10, and neurofilament light chain show strong associations with both AD and MD, independent of chronological age and frailty status [21]. These data suggest that immune dysfunction interacts with vascular and neurodegenerative processes to determine dementia trajectories. Moreover, genetic studies have revealed that chemokine receptor 5 (CCR5) deficiency enhances neuronal vulnerability to oxidative stress and synergizes with the ApoEε4 allele to increase the risk of vascular and mixed forms of dementia [9]. ApoEε4, the strongest common genetic risk factor for late-onset AD [22], also promotes cerebrovascular dysfunction, including BBB breakdown, small-vessel injury and worse outcome after stroke [23]. Together, these mechanisms and epidemiological data show that APOE4 increases the chance of coexisting AD and vascular pathology, thereby accelerating cognitive decline in mixed dementia.

Collectively, these findings outline a complex, multifactorial etiology for mixed dementia, in which chronic cerebral hypoperfusion, oxidative and inflammatory stress, and barrier dysfunction interact to amplify neurodegeneration. Understanding the interplay between vascular and neurodegenerative mechanisms is thus essential for identifying effective, multimodal therapeutic strategies capable of addressing the heterogeneity of late-life dementia.

## 2. Therapeutic Drugs

Established drug therapies for AD, such as cholinesterase inhibitors and memantine, do not modify the disease course and provide only modest clinical benefits. Two monoclonal antibodies targeting the Aβ protein (aducanumab and lecanemab) have been approved in the United States, and two agents (lecanemab and donanemab) have been recently approved by the Therapeutic Goods Administration in Australia. Clinical trials have demonstrated that monoclonal antibodies are effective at removing amyloids from the brain in individuals with early AD. Cognitive benefits are statistically significant, but do not achieve the minimal clinically important difference [2].

It must be emphasized that amyloid-related imaging abnormalities of vasogenic edema and microhemorrhages occur more frequently in response to antibody treatment. Although these events are usually asymptomatic or transient, in some people they are serious or fatal. Cerebral amyloid angiopathy (CAA) often accompanies dementia-associated pathologies and is important in the context of anti-amyloid monoclonal therapies due to the associated risk of hemorrhage [24].

Targeting amyloid as a unimodal strategy is unlikely to be sufficient and future therapies may need to be multimodal, targeting multiple pathogenic pathways. Since the burden of dementia is greatest in the older population, where mixed dementia pathology dominates, identifying effective therapies for this group is a challenge [2]. Cholesterol-lowering agents, such as statins, which are commonly used in patients with vascular diseases and dyslipidemia, may affect the progression of VaD. Several previous studies highlighted the potential therapeutic efficacy of statins in treating VaD [25]. Moreover, some patients with AD or mixed dementia with indication for lipid-lowering medication may benefit cognitively from statin treatment; however, further research is needed to clarify the findings of sensitivity analyses [26].

Since oxidative stress is considered a shared mechanism that accelerates both neurodegeneration (Alzheimer’s pathology) and vascular injury, determining mixed dementia, antioxidants (e.g., vitamins E and C, polyphenols) have been tested, but large clinical trials show limited or inconsistent benefits [27,28]. Current research is shifting toward targeting mitochondrial dysfunction, Nrf2 activation (an antioxidant response pathway), and lifestyle interventions (exercise, diet) that reduce oxidative load. Nrf2 is a major transcriptional factor that controls gene expression under both normal health and pathological conditions. It regulates and controls key processes related to oxidative stress, neuro-inflammation, autophagy, and mitochondrial bioenergetics in the central and peripheral tissues. Decreased expression of Nrf2 and its downstream target genes was identified in AD. Recent studies have shown that Nrf2 interferes with various main pathogenic processes in AD, including amyloid and tau pathologies [29].

Studies have confirmed that Nrf2/antioxidant response element (ARE) signaling pathway plays a vital role in antagonizing chronic cerebral hypoperfusion injury. Imperatorin, a coumarin derivative, has confirmed to exert antioxidant effects in VaD via the Nrf2 signaling pathway. In a VD cell model, this treatment increased the mitochondrial membrane potential of hippocampal neurons, reduced ROS level and oxidative stress damage and inhibited hippocampal neuroapoptosis [30].

Other Nrf2 activators have been extensively studied in preclinical settings; however, it is challenging to determine which compounds should advance to clinical trials in Vascular Contributions to Cognitive Impairment and Dementia (VCID). Therapeutically, Nrf2/mitochondrial targets and vascular-first strategies are biologically plausible, but large, positive clinical trials in mixed dementia are still lacking. Since no Nrf2 activators are currently being trialed in VCID studies, patient selection, drug dose, delivery modalities, timing, and administration protocol need to be developed and optimized to maximize therapeutic effects while avoiding potential side effects [13].

## 3. Future Direction: New Therapeutic Strategies

Despite advances in understanding neurodegenerative disease mechanisms, effective treatments remain elusive. Based on the premise that oxidative stress is the mechanism that, linking AD and VaD, leads to MD, we sought to identify the most relevant molecular targets involved in the redox state, and therapeutic approaches that could broadly modulate these mechanisms. Here, we reviewed recent literature (2020–2025) reporting the use of delivery systems for antioxidant proteins and/or miRNAs that regulate the redox state. This research revealed that EVs are the delivery tool that can reach the affected brain tissue carrying a complex cargo characterized by molecules that modulate the redox state.

To this end, new therapeutic targets can be identified through high throughput screening setups based on preclinical studies, including both in vitro and vivo models.

To mimic AD in vitro, neuron cells may be treated with Aβ oligomers, tau aggregates, or LPS whereas VaD can be modeled by exposing cells to oxygen/glucose deprivation, hypoxia–reoxygenation, or hydrogen peroxide treatment [31]. By combining the two, a model of MD can be produced, allowing assessment of cellular responses to these insults, and investigations on neuron–glial–astrocyte interaction through co-culture systems [32].

Complementing these in vitro approaches, in in vivo models, such as bilateral carotid artery stenosis (BCAS) in mice, effectively reproduce VaD pathology [33]. BCAS combined with AD transgenic lines (e.g., 5xFAD) have successfully established a novel experimental model of MD, recapitulating both vascular and amyloid pathologies are modeled, providing a valuable platform to study mixed dementia mechanisms and potential therapeutic strategies [1].

Within experimental settings, extracellular vesicles (EVs), a range of membrane-enclosed vesicles encompassing exosomes, apoptotic bodies, and autophagic vesicles, have emerged as key mediators of intercellular communication in the central nervous system (CNS), and are increasingly recognized for their involvement in the pathogenesis of neurodegenerative disorders like Alzheimer’s disease, Parkinson’s disease, amyotrophic lateral sclerosis, multiple sclerosis and Huntington’s disease. In vivo studies demonstrate EVs’ crucial role in maintaining CNS homeostasis, modulating neuroinflammatory responses, and influencing tissue repair and regeneration following injury, thereby impacting disease progression and recovery. Among them, neuron-derived exosomes/EVs (NDEs) exhibit neuroprotective effects by promoting Aβ clearance, modulating tau pathology, and reducing inflammation. However, exosomes can also contribute to disease propagation, for instance by spreading pathogenic tau or mediating complement-dependent neurotoxicity [34]. Their unique properties, including small size and ability to cross the BBB, position them as promising candidates for biomarkers in CNS diseases [35]. Collectively, EVs play both protective and detrimental roles in neurodegenerative diseases.

Mesenchymal stem cells (MSCs) have become a focal point in medical research. Increasing evidence suggests that MSCs do not solely operate through cell differentiation but also mediate a myriad of biological effects through their secreted extracellular vesicles (MSC-EVs). Moreover, MSC-EVs offer several advantages over MSCs, including enhanced targeted delivery, reduced immunogenicity, and enhanced reparative potential. Consequently, they represent a promising alternative for developing novel therapeutic strategies [36]. MSC-EVs and MSC-derived exosomes (MSC-Exos) are small vesicles packed with bioactive substances, including proteins and nucleic acids that exhibit potent anti-inflammatory properties and immunomodulatory effects. These properties make them highly promising for addressing inflammatory and autoimmune disorders [37] and may potentially be leveraged in neurodegenerative diseases, where neuroinflammation plays a central role. Additionally, MSC-EVs can be engineered for targeted drug delivery, enhancing their potential for clinical application [38].

There is growing evidence that MSC-EVs and MSC-Exos can significantly improve the curative effect of oxidative stress-related diseases by reducing oxidative and inflammatory markers in various systemic diseases and mitigating apoptosis and vascular injury induced by oxidative stress. Numerous experimental studies have shown that both local and systemic administration of these vesicles effectively inhibits the oxidative stress response in diseases and promotes the survival and regeneration of damaged parenchymal cells. The mRNA and miRNAs contained in MSC-EVs and MSC-Exos are the most important bioactive molecules in disease treatment, as they inhibit the apoptosis, necrosis and oxidative stress across multiple tissues, such as lung, heart, kidney, liver, bone, and skin, promoting their survival and regeneration [39].

MSC-EVs have been demonstrated therapeutical too, as shown in Table 2. MSC-Exos, such as from human umbilical cord MSC (hUC-MSC), significantly decreased the LPS- or H_2_O_2_-induced oxidative stress and the expression of pro-inflammatory cytokines (IL-6 and TNF-α) in vitro, while promoting an anti-inflammatory (classical M2) phenotype in an LPS-treated mice. Mechanistically, these exosomes upregulated Nrf2 and inhibited the LPS-induced NF-κB p65 phosphorylation and NLRP3 inflammasome activation. Notably, inhibition of Nrf2 with ML385 abolished the anti-inflammatory and antioxidative effects of the exosomes [40].

hUC-MSC-derived EVs (hUC-MSC-EVs) exhibited therapeutic potential in VaD by activating the PI3K/AKT/Nrf2 pathway. In a VaD rat model, hUC-MSC-EVs administration mitigated neurological impairment, improved cognitive function, and restored brain tissue structure. These protective effects were associated with reduced microglial M1 polarization, inflammation, and oxidative stress. hUC-MSC-EVs activated the PI3K/AKT/Nrf2 pathway in brain tissues, highlighting its crucial role in mediating the observed neuroprotection. When the PI3K pathway was inhibited, the beneficial effects of hUC-MSC-EVs on microglial polarization, inflammation, and oxidative stress were partially reversed, emphasizing the significance of the Nrf2 pathway in hUC-MSC-EVs-mediated neuroprotection in VaD [41].

It has been demonstrated that hUC-MSC-Exos exhibit neuroprotective effects following Traumatic Brain Injury (TBI) by engaging the lncRNA TUBB6/Nrf2 pathway. Administering hUC-MSC-Exos to a TBI mouse model reduces TBI-induced inflammation and ferroptosis, while enhancing lncRNA TUBB6 expression. This upregulation facilitates Nrf2 nuclear translocation, aiding in the mitigation of TBI-induced neuronal death. Furthermore, hUC-MSC-Exos suppress ferroptosis-related markers such as ACSL4, and their modulation of the Nrf2 signaling pathway contributes significantly to alleviating oxidative damage and inflammation associated with TBI. These findings underscore the therapeutic potential of hUC-MSC-Exos in managing TBI-related neurological complications [42].

Other perinatal sources of MSCs have demonstrated antioxidant potential: human amniotic fluid (AF) and amniotic fluid stem cells (AFSCs) represent a feasible source of EVs to counteract oxidative damage in target cells, as demonstrated in 2024 by Bollini’s group [43] and by our lab [44] in skeletal and cardiac muscle injury and cancer disease, respectively. Moreover, we demonstrated the ROS-modulating effects of these extracellular vesicles in an AD in vitro model, proposing AFSC-EV as a therapeutic tool to stop AD progression [45,46]. Although there is limited research on the use of AFSC-EVs for VaD and vascular diseases, studies have demonstrated the successful application of AF-EVs and AFSC-EVs in therapy, harnessing their anti-inflammatory, angiogenic and regenerative properties [47].

These studies propose a more targeted therapeutic approach towards the modulation of the intracellular redox state of nervous tissue to simultaneously target the elements common to pathologies that lead to neurodegeneration. In particular, Nrf2 impairment permits both neurodegenerative ROS accumulation (from Aβ/tau) and vascular oxidative injury to proceed unchecked—producing additive damage to lipids, proteins, mitochondria and cellular energy metabolism. MSC-derived EVs or engineered EVs carrying Nrf2 mRNA/protein or miRNAs that disinhibit Nrf2 (e.g., targeting KEAP1 or p62-KEAP1 interactions) are attractive because EVs can cross the BBB and deliver complex cargos to neurons/glia/endothelia [8].

Here, we show a focused, literature-backed table (Table 3) summarizing miRNAs and proteins reported in mesenchymal stem cell (MSC)-derived EVs that have been linked to activation/promotion of the Nrf2 antioxidant pathway (either by directly targeting Nrf2 regulators, delivering antioxidant enzymes, or inducing Nrf2/HO-1/NQO1 expression in recipient cells). The number of well-characterized cargos specifically promoting Nrf2 in MSC-EVs is limited; some entries are indirect and infer the role via Nrf2 activation.

Notably, certain miRNAs (miR-21, miR-194, miR-200a) have direct mechanistic links to Nrf2 regulation (PTEN → PI3K/Akt; Bach1 repression; Keap1 targeting). Several studies demonstrate that MSC-EVs transfer these miRNAs and that knockdown/inhibition of either the miRNA or Nrf2 reduces the EVs protective effect, supporting causality [48,49].

Antioxidant enzymes (SOD1, GPX1, catalase, thioredoxin) are frequently detected in MSC-EVs proteomes and can immediately reduce ROS levels upon transfer; this lower ROS milieu facilitates Nrf2-mediated transcriptional recovery in recipient cells [50,51].

However, evidence levels vary in several aspects. Some papers rely on proteomic/miRNA profiling to show the presence of these cargos, whereas others demonstrate functional Nrf2 activation in recipient cells using in vitro (OGD/R or H_2_O_2_-induced injury) or animal models. A few studies use engineered EVs to deliver Nrf2 directly.

**Table 3:** Table summarizing miRNAs and proteins linked to antioxidant effect or/and promotion of the Nrf2 pathway that have been identified in mesenchymal stem cell (MSC)-derived EVs. The source of MSC, the context and the mechanism involved are specified. → = produce the effect.

Molecule Type	Molecule	Source (MSC-EV Context)	Mechanism	References
miRNA	miR-21 (miR-21-5p)	human placenta MSCs-derived exosomes; human amniotic fluid cells- EVs (hAFSC-EVs) → anticancer	Downregulates PTEN → activates PI3K/Akt signaling → promotes Nrf2 nuclear translocation and antioxidant responses	[44,48]
miRNA	miR-29b-3p	EVs from IFN-γ-primed mouse bone marrow cells MSCs → astrocytes; hAFSC-EVs → anticancer	miR-29b-3p targets the downstream inhibitor Bach2 → which thus lifts repression on Nrf2 axis	[44,52]
miRNA	miR-100-5p	EVs from MSCs in Parkinson’s disease model Wharton’s jelly MSC-MVs → anticancer	miR-100-5p from MSC-EVs promotes dissociation of Nrf2 from Keap1 → Nrf2 activation, antioxidant enzyme induction	[49,53,54,55]
miRNA	miR-124	EVs from MSCs → neurons (spinal cord injury model)	miR-124 delivered in MSC-EVs stabilizes the p62–Keap1–Nrf2 loop, promotes Nrf2 nuclear translocation and reduces ROS	[56]
miRNA	miR-125b-5p	EVs from Wharton’s jelly MSC or ADSC → traumatic wound modelMSC-EVs → endothelial cells ferroptosis in lung sepsis	Promotes endothelial repair/angiogenesis and reduces oxidative stress: modulation of NFκB axis miRNA targets Keap-1, thereby promoting Nrf2 activation	[49,57,58]
miRNA	miR-146a	human adipose MSC-EVs → in senescent endothelial cells	mitigate oxidative stress	[59]
miRNA	miR-194	MSC-Exos → downregulation of ferroptosis in OGD/R injury	Targets Bach1 (a transcriptional repressor of HO-1), thereby disinhibiting Nrf2/HO-1 signaling	[60]
miRNA	miR-200a-3p	EVs from MSCs in kidney injury model and in diabetic rats, hepatic fibrosis	miR-200a-3p from MSC-EVs activates KEAP1–Nrf2 signaling (decreased Keap1, increased Nrf2)	[49,61,62,63]
miRNA	miR-210	Wharton jelly-derived MSC-EVs enriched with miR-210 in damaged renal cells	Reducing apoptosis and ROS accumulation	[64]
Protein	IGF-1 (insulin-like growth factor 1)	EVs from human umbilical cord MSCs (hUC-MSC-EVs) in ovarian insufficiency model	EV-encapsulated IGF-1 activates Nrf2/HO-1 signaling in recipient granulosa cells (nuclear Nrf2 ↑)	[65]
Protein	GPX1 (glutathione peroxidase 1)	MSC exosomes in Rescuing Renal Injury	Reduces hydrogen peroxide → contributes to intracellular redox re-balancing and supports Nrf2-dependent restoration of antioxidant capacity after EV uptake.	[50]
Protein	Catalase (CAT)	hAFSC-EVs in cardiac injury	Detoxifies H_2_O_2_ → lowers oxidative stress in recipient cells; presence/function reported in EV studies and contributes to net Nrf2 pathway benefits.	[43]
Protein	Thioredoxin	hUC-MSC-Exos in doxorubicin-induced cardiotoxicity and intervertebral disc degenerationhAFSC-EVs → anticancer	anti-ferroptosis process	[44,66,67]
Protein	SOD1 (superoxide dismutase 1)	hAFSC-EVs in different models	EV-delivered SOD1 provides enzymatic dismutation of superoxide → reduces ROS burden and supports Nrf2-mediated antioxidative recovery in recipient cells.	[43,44,45]
Protein	Nrf2 protein itself	EVs from H_2_S-preconditioned MSCs (H_2_S-EVs) delivered to neurons	Free Nrf2 packaged into EVs (Via HSP70/LAMP2A recognition) increasing mitochondrial Nrf2 accumulation and antioxidant/mito-homeostasis effects	[68]

## 4. Conclusions

Oxidative stress is a central contributor to both Alzheimer’s and vascular dementia. In mixed dementia, it acts as a common pathway that worsens both vascular damage and neurodegeneration, making it a key factor in disease progression. Since current therapies are not sufficient to counteract these pathologies—either individually or in combination—and classical antioxidant treatments have shown limited efficacy, we sought to identify the most relevant molecular targets involved in redox regulation, as well as therapeutic strategies capable of broadly modulating these mechanisms. Our investigation indicates that extracellular vesicles represent a promising delivery system capable of reaching affected brain regions while carrying a complex cargo of molecules that modulate the redox state. Collectively, these studies suggest a more targeted therapeutic approach aimed at adjusting the intracellular redox balance in neural tissue, with the goal of simultaneously addressing the shared mechanisms underlying various neurodegenerative conditions, and combining Nrf2 activation with vascular protection (blood pressure control, antiplatelet/antithrombotic where appropriate), anti-amyloid/pro-tau strategies and mitochondrial support to address both arms of mixed dementia. While direct evidence linking perinatal MSC-EVs to vascular disease therapy is restricted, their properties suggest a promising avenue for future research. Exploring their potential in preclinical models of combined neuro-vascular and neurodegenerative diseases could provide valuable insights into their therapeutic applications and continued research may establish EV/exosomes as a transformative approach in AD and/or VaD therapy.

Overall, despite these advancements, clinical translation requires a deeper understanding of exosome/EVs biology, improved isolation techniques, and personalized strategies, as often stated in the literature [69].

## Figures and Tables

**Table 1:** Comparative Table summarizing the shared and distinct oxidative stress biomarkers that are elevated in Alzheimer’s disease, Vascular dementia, and Mixed dementia. ↑ = increase; ↓ = decrease.

Biomarker Category	Specific Markers/Assays	Alzheimer Disease	Vascular Dementia	Mixed Dementia	Key Refs
Lipid peroxidation	MDA (malondialdehyde), 4-HNE (4-hydroxy-2-nonenal) adducts, F_2_-isoprostanes	↑ MDA, 4-HNE, isoprostanes in brain tissue, CSF and blood	↑ MDA/isoprostanes. measurable peripherally	Typically ↑, reflecting additive pathology (AD neurodegeneration + vascular oxidative injury).	[14]
Protein oxidation/nitrosative damage	Protein carbonyls, 3-nitrotyrosine, methionine sulfoxide	↑ protein carbonyls and nitrotyrosine in brain and CSF linked to tau/Aβ pathology.	↑ protein oxidation reported in VaD (secondary to ischemia).	Mixed cases show markers of both processes (neurodegenerative nitrosative damage + vascular ischemic protein oxidation).	[15]
Antioxidant enzyme levels (enzymatic activity)	SOD (Cu/Zn and Mn), Catalase (CAT), Glutathione peroxidase (GPx), glutathione (GSH)	Often ↓ CAT and ↓/variable GPx; SOD findings variable	Decreased antioxidant defenses reported (↓ GPx, ↓ SOD/CAT in some studies),	Reduced antioxidant capacity generally, pattern depends on relative burden of vascular vs. AD pathology—often intermediate or additive deficits.	[16,17]
Non-enzymatic antioxidants/redox status	Total antioxidant capacity, reduced GSH, thiol levels	↓ GSH/↓ total antioxidant capacity in brain and periphery	↓ GSH/↓ antioxidant capacity reported in VaD	↓, often reflecting contributions from both pathologies.	[18]
Mitochondrial function—bioenergetics	ETC complex activities (I, III, IV), ATP levels, oxygen consumption rate	↓ ETC activity (esp. complex IV), reduced ATP production, impaired mitochondrial dynamics and mitophagy.	Mitochondrial dysfunction in chronic cerebral hypoperfusion/ischemia models—decreased ATP, altered membrane potential.	Features of both: AD-type OXPHOS impairment plus ischemia-driven mitochondrial deficits—net worse bioenergetic failure.	[19]
Mitochondrial genome/markers	mtDNA deletions, mtDNA copy number, oxidative mtDNA damage (8-OHdG)	↑ mtDNA damage/deletions and altered copy number reported in AD brain.	mtDNA damage reported in brains exposed to ischemia/vascular pathology.	Combined mtDNA damage patterns expected; mixed dementia may show greater overall mtDNA damage due to dual insults.	[19,20]

**Table 2:** Table summarizing studies in vitro and/or in vivo in which oxidative stress was modulated by mesenchymal stem cell (MSC)-derived EVs. The source of MSC, the context and the mechanism involved are specified. ↑ = increase; ↓ = decrease.

Study/Source	Model (In Vitro/In Vivo)	Stem Cell/EV Type	Main Effects/Mechanisms
LPS or H_2_O_2_ stimulation; LPS-treated mice [40]	In vitro (LPS or H_2_O_2_-induced oxidative stress) and In vivo (LPS-treated mice)	hUC-MSC-Exos	↓ Oxidative stress; ↓ IL-6, TNF-α; promotes M2 phenotype; ↑ Nrf2 activation; ↓ NF-κB p65 phosphorylation; ↓ NLRP3 inflammasome; Nrf2 inhibition abolishes effects.
VaD rat model [41]	In vivo	hUC-MSC-EVs	Activates PI3K/AKT/Nrf2 pathway; improves cognitive function; restores brain tissue; ↓ M1 microglia; ↓ inflammation and oxidative stress; inhibition of PI3K reduces benefits, showing Nrf2 dependence.
Traumatic Brain Injury (TBI) mouse model [42]	In vivo	hUC-MSC-Exos	Engages lncRNA TUBB6/Nrf2 pathway; ↑ Nrf2 nuclear translocation; ↓ inflammation; ↓ ferroptosis (↓ ACSL4); protects neurons and reduces oxidative damage.
Skeletal and cardiac muscle injury [43]	In vitro (target cells) and In vivo (injury models, as reported)	AF-EVs	Antioxidant effects; protection of skeletal and cardiac muscle; ↓ oxidative damage.
Cancer disease model [44]	In vitro	AFSC-EVs	Protection from oxidative stress; ROS-modulating activity.
Alzheimer’s disease (AD) model [45,46]	In vitro	AFSC-EVs	ROS modulation; proposed therapeutic tool to halt AD progression.
Applications in vascular diseases and VaD (general evidence) [47]	In vivo (reported across studies) and In vitro	AF-EVs and AFSC-EVs	Anti-inflammatory, angiogenic, regenerative properties; therapeutic potential suggested though limited data for VaD specifically.

## Data Availability

No new data were created or analyzed in this study.
